# Isolation of cancer-derived extracellular vesicle subpopulations by a size-selective microfluidic platform

**DOI:** 10.1063/5.0008438

**Published:** 2020-06-08

**Authors:** Zheyuan Chen, Yi Yang, Hirohito Yamaguchi, Mien-Chi Hung, Jun Kameoka

**Affiliations:** 1 Department of Electrical and Computer Engineering, Texas A&M University, College Station, Texas 77840, USA; 2 Department of Molecular and Cellular Oncology, The University of Texas MD Anderson Cancer Center, Houston, Texas 77030, USA; 3 Cancer Research Center, Qatar Biomedical Research Institute, College of Health and Life Sciences, Hamad Bin Khalifa University, Qatar Foundation, Doha, Qatar; 4 Graduate Institute of Biomedical Sciences and Center for Molecular Medicine and Office of the President, China Medical University, Taichung 404, Taiwan; 5 Department of Biotechnology, Asia University, Taichung 413, Taiwan; 6 Department of Material Science and Engineering, Texas A&M University, College Station, Texas 77840, USA

## Abstract

Extracellular vesicles (EVs) play an important role in intercellular communication. Recently, there has been increasing interest in EVs as potential diagnostic biomarkers and therapeutic vehicles. However, the molecular properties and cargo information of EV subpopulations have not yet been fully investigated due to lack of reliable and reproducible EV separation technology. Current approaches have faced difficulties with efficiently isolating EVs from biofluids, especially subpopulations of small EVs. Here, we report an EV isolation method based on a size-selective microfluidic platform (ExoSMP) via nanomembrane filtration and electrophoretic force. This unique platform offers an enhanced approach to sorting a heterogeneous population of EVs based on size, with the additional advantages of being label-free and low-cost, and featuring a short processing time (<1 h), and convenient integration with downstream analysis. In this research, we used ExoSMP to demonstrate the isolation of cancer-derived small EVs (30–120 nm) with high recovery (94.2%) and reproducibility at an optimum sample flow rate. Furthermore, we investigated isolation of EV subpopulations by altering nanomembrane combinations with different pore size combinations (50 and 100 nm, 30 and 100 nm, 30 and 200 nm, and 30 and 50 nm). This ExoSMP technique can serve as a standardized EV isolation/separation tool, facilitating the clinical prospects of EVs and opening up a new avenue for future point-of-care applications in liquid biopsies.

## INTRODUCTION

Among translational and clinical researchers, there is significant interest in extracellular vesicles (EVs) as potential diagnostic biomarkers and therapeutic vehicles that can be fast-tracked to clinical evaluation and precision medicine applications.^1,2^ EVs refer to a heterogenous lipid bilayer of membranous structures that hold information in the form of proteins, lipids, or nucleic acids, thereby physiologically and pathologically influencing the intercellular communication of both the recipient and parent cells.^3^ EVs are released by most viable cells. These particles can be isolated and collected from various bodily fluids such as blood, saliva, urine, lymph, and milk. In the past few decades, there are at least three main subgroups that have been identified and termed as exosomes, microvesicles, and apoptotic bodies.^4,5^ The existing criteria adopted to discriminate among the subpopulations are based on size, density, function, and molecular cargo.^6^ The physical properties of EVs contribute to distinct biological functions and organ distribution patterns. A unique cancer-derived EV population was recently identified and termed “large oncosomes” due to their atypical size. They transport oncogenic materials and are more specific to cancer cells.^7^ Exosomes are nano-sized populations of EVs that are released by most viable cells. Two exosome subpopulations were recently identified as large exosome vesicles (Exo-L, 90–120 nm) and small exosome vesicles (Exo-S, 60–80 nm), and a population of non-membranous nanoparticles was newly discovered and termed “exomeres” (∼35 nm).^8^ Proteomic profiling of these three nanoparticle subsets has revealed that each subgroup contains unique protein cargoes. These proteins participate in different signaling pathways and have distinct biological functions.^8^ Increasing evidence has revealed the underlying heterogeneous nature of exosomes within intensively distinct particulate secretomes.^9^ EVs display a diverse range of sizes in terms of diameter, but the terms of exosomes and microvesicles tend to cause inaccuracy and confusion by both manifold. Therefore, the three main subgroups of EVs can be categorized based on size in this study: small EVs (<120 nm, sEVs), medium EVs (120 nm–200 nm, mEVs), and large EVs (>200 mm, lEVs). Tremendous attention has recently been focused on sEVs, which play a vital role in tumorigenesis, tumor microenvironments, cancer metastasis, and chemotherapeutic resistance.^8,10,11^ Thus, the molecular properties and cargo information of various subpopulations of EVs must be dissected in order to investigate their clinical potential as diagnostic, prognostic, and therapeutic candidates in liquid biopsies.

To unravel the mystery of EVs, several conventional approaches have been developed such as ultracentrifugation (UC),^12–14^ precipitation,^15–17^ and membrane filtration,^18–20^ and immunoaffinity-based separation.^21^ UC is currently the most common approach for isolating EVs. This gold standard method differentiates among sizes with a sequence of centrifugations from low to high rotation speeds. The key barriers to implementing this approach in a clinical setting are the lengthy operating time (more than 5 h), low EV recovery, purity, and reproducibility, and poor specifications. There is also evidence that the high centrifugal force (100 000–200 000 g) can cause EV fusion and coagulation, and may damage their structure, properties, and function.^22,23^ Precipitation approaches have drawbacks such as small sample volumes and low purity. The polymer matrix used for precipitation can influence the biological activity of the EVs. Therefore, precipitation approaches have some difficulty in isolating intact EVs for cancer research and biomarker discovery.^15,16^ The challenges accompanying membrane-based filtration include difficulties with operation optimization, low specificity, and poor reproducibility due to membrane fouling.^24^ A major drawback of immunoaffinity-capturing methods is the low yield due to limitations in specific EV–antibody interactions.^21^ This also requires pre-treatment and lengthy processing times. Size-selective EV isolation has also been demonstrated by the asymmetric flow field-flow fraction (AF4) method^8^ and multiple nanomembrane filter devices.^25^ These two approaches have been proven to further isolate EV subgroups based on size. However, the equipment for AF4 is expensive, not user friendly, and bulky, and the method requires a lengthy processing time. The drawback of multiple nanomembrane filtration is the irregular liquid pressure distribution through five nanomembranes (the first to fifth membrane pressure applications are not same), which reduces reproducibility. Other approaches such as nanowire trapping,^26,27^ acoustic separation,^28,29^ lateral displacement,^30,31^ viscoelastic flow separation,^32^ electrophoretic separation,^33^ and dielectrophoretic separation^34^ have been implemented. However, these methods are unable to efficiently separate EVs based on the size of the subpopulation and suffer from limitations such as additional reagents/labels, pre-treatment steps, lengthy processing times, and low reproductivity, yield, EV recovery, and purity.

In this study, we demonstrated EV isolation on a single size-selective microfluidic platform (ExoSMP) that targets an automated, consistent, and reliable isolation method. This is based on a size-selective process accomplished via nanomembrane filtration and the hydrodynamic properties of nanoparticles. This unique platform enables the identification and harvesting of size-specific EVs (i.e., sEVs and mEVs) with the additional advantage of being label-free and featuring low cost, short processing times, and convenient integration with other on-chip technology and downstream analysis. Below, we first demonstrate intact sEV isolation based on size from mEVs, lEVs, proteins, and other bio-fragments with a short processing time of less than 1 h. We then exhibit the isolation of subpopulations of EVs by simply altering the pore sizes of nanomembrane filters. Quantitative analysis of the isolated EVs by nanoparticle tracking analysis (NTA) is shown to confirm high recovery from the cancer cell-derived EVs.

## RESULTS AND DISCUSSION

### Design and working principle of ExoSMP

**FIG. 1.**
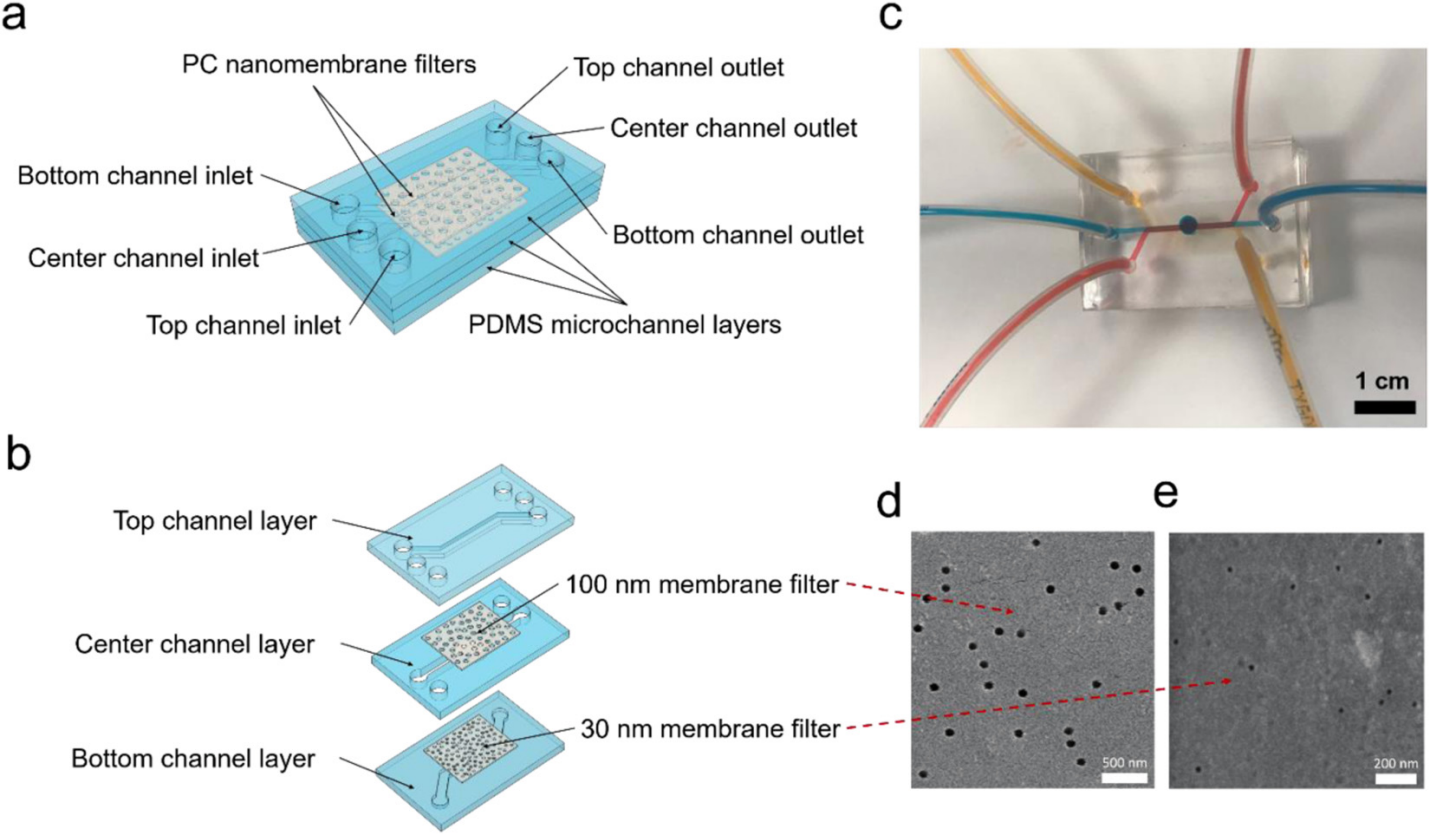
Schematics of the size-selective microfluidic platform device. (a) Schematic diagram of the three-layer microfluidic device. (b) 3D explosive view of the microfluidic device. The nanomembrane filters are sandwiched by three microchannel layers. (c) An optical image of the microfluidic device with 30 and 100 nm membrane filters. De-ionized (DI) water with color dyes was pumped into the top (red), center (blue), and bottom (orange) microchannels, respectively. SEM images of the nanomembrane filters with pore sizes (in diameter): (d) 100 nm and (e) 30 nm.

As shown in Figs. 1(a), 1(b), and S1 in the supplementary material, the microfluidic platform consists of three horizontally aligned microchannels and two polycarbonate (PC) nanoporous membranes (with pore sizes of 30 and 100 nm) sandwiched between the microchannels. A large through-hole is punched in the center microchannel in order to form an enlarged fluid exchange area through the top to the bottom microchannels. Gold electrodes are connected to the top and bottom microchannels to induce the electrophoretic force through the two nanoporous membranes. An optical image of the microfluidic platform is presented in Fig. 1(c). The scanning electron microscopy (SEM) images of the 30 and 100 nm nanoporous membranes are shown in Figs. 1(d) and 1(e), respectively. The entire sample solution is pumped into the top microchannel (red) through the top inlet, and the extraction buffer solutions phosphate-buffered saline (PBS) are pumped into the center (blue) and bottom (yellow) microchannels at optimum flow rates.

The flow direction in the microchannels is perpendicular to the vertical electrophoretic force acting through the top to bottom microchannels. Less particle fouling can be expected using ExoSMP because the transportation direction of the sample flow and electrophoretic force are perpendicular, as compared to other membrane-filtration approaches.^18–20^

**FIG. 2.**
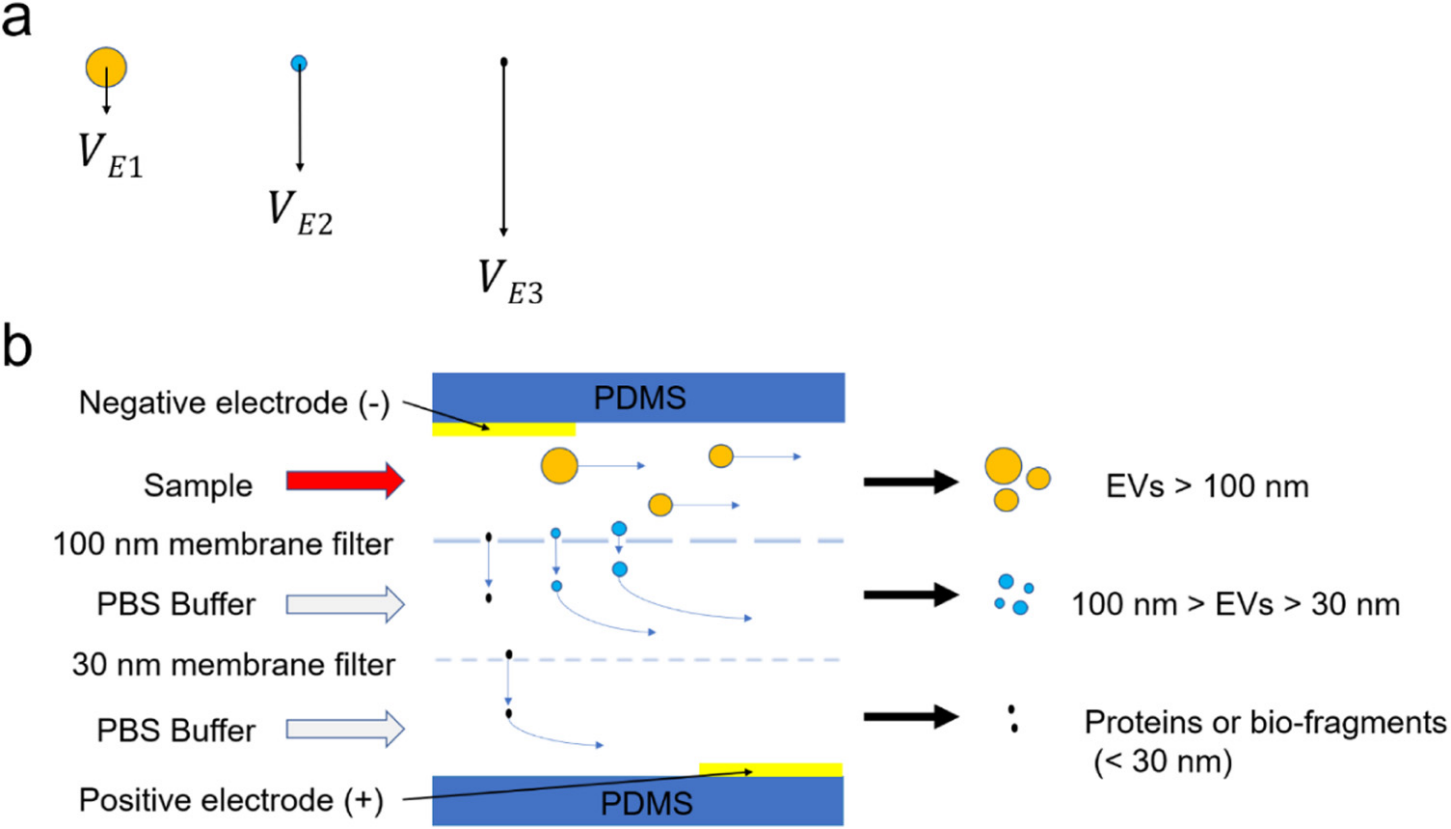
Schematic diagrams for theory and working principle of the ExoSMP device. (a) Negatively charged particles with different dimensions are subject to the electrophoretic force by the applied electric field in vertical direction. They have different vertical velocity profiles due to their dimensions. (b) Working principle of size-selective isolation of EVs using the ExoSMP device with 30 and 100 nm membrane filters in side view. Schematic particle trajectories are labeled in blue arrows.

In the present study, there were two perpendicular forces on the particles: fluidic flow and electrophoretic force [see Fig. 2(a)]. The fluidic flow was induced in the horizontal direction, while the electrophoretic force was prompted by the applied electric field in a vertical direction. The forward velocity ($V_{F}$) was equal to the flow rate of the fluid, while the electro-elastic lift velocity ($V_{E}$) was calculated based on Eq. (1) under a laminar flow condition,^35^

$$V_{E}=\frac{QE}{6\pi \mu R},$$ (1)

where *Q* is the surface charge of the particle, *E* is the applied electric field, *μ* is the dynamic viscosity of the fluid, and *R* is the hydrodynamic radius of the particle. With the applied voltage and fluid, the electrophoretic velocity was, therefore, inversely proportional to the radius and surface charge of the particle. According to the Grahame equation, the zeta potential of the particle could be converted to the surface charge,^36^ as shown in Eq. (2),

$$\;\;\;Q=\frac{\varepsilon \cdot \zeta }{\lambda },$$ (2)

where ε is the dielectric constant, and ζ and λ are the zeta potential and Debye screening length, respectively. The zeta potential of the proteins was approximately two times larger than that of the EVs at their maximum.^26,37,38^ Thus, the most dominant factor for determining the electrophoretic velocity was the hydrodynamic radii of the proteins (5–10 nm) and sEVs (30–100 nm).

Particles of various sizes under fluidic flow have the same forward fluidic velocity ($V_{F}$). However, particles with smaller hydrodynamic radii (R) tend to have larger electrophoretic velocity. The variables $R_{1}$, $R_{2}$, and $R_{3}$ represent particles with three different hydrodynamic radii. If $R_{1}>R_{2}\gg R_{3}$, we have $V_{E1}<V_{E2}\ll V_{E3}$ according to Eq. (1), as shown in Fig. 2(a). Smaller dimensional particles tend to have faster vertical motion. That is to say, the vertical velocity of the proteins was much faster than that of the EVs under the applied electric field. Moreover, the vertical electrophoretic velocity of the larger EVs was much slower than that of the smaller EVs and proteins, thereby enabling the proteins and smaller EVs to pass vertically through the nanoporous membrane.

The workflow of the EV isolation is shown in Fig. 2(b). EVs, including sEVs, mEVs, and lEVs, are negatively charged particles.^26,39^ ExoSMP is able to separate particles based on the pore sizes of nanomembrane filters and hydrodynamic properties of the particles. Particles larger than the pore size are retained by the fluidic force in the same microchannel. Negatively charged particles such as apoptotic cells and microvesicles are attracted to the cathode but retained in the top channel outlet due to their dimensions and slow vertical velocity. Positively charged molecules such as certain proteins are retained and attracted to the gold anode electrode located on the top microchannel. Nanovesicles and negatively charged proteins can pass vertically through the 100 nm nanoporous membrane due to electrophoretic force. EVs with dimensions between 30 and 100 nm are retained in the center microchannel and collected at the center channel outlet. Negatively charged proteins are quickly guided into the bottom microchannel through the 30 nm membrane filter.^40^

### EV isolation at different sample flow rates

**FIG. 3.**
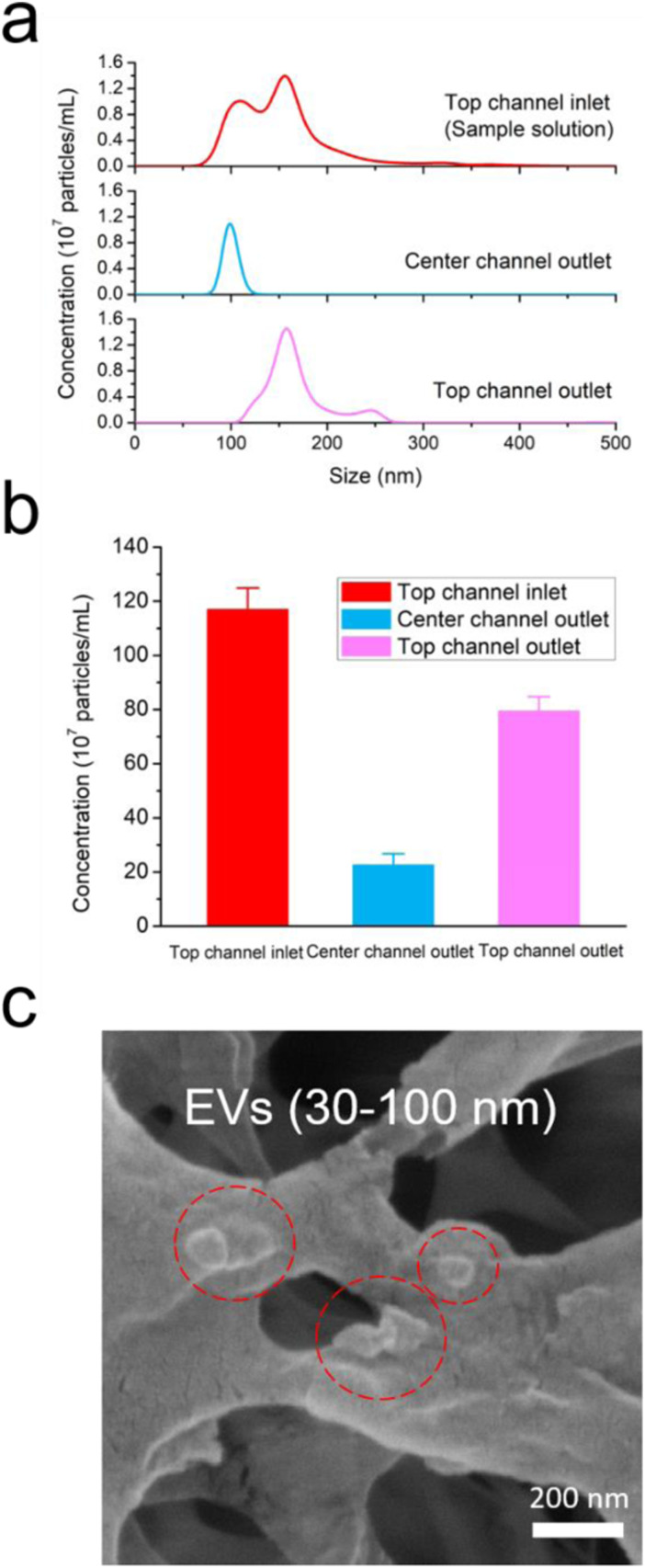
Isolation of EVs from cancer cell derived EVs with 30 and 100 nm nanoporous membranes at the sample flow rate of 10 *μ*l/min. (a) The size distributions of the particles in top channel inlet (original sample solution, red line), center channel outlet (blue line), and top channel outlet (pink line) solutions. (b) The total numbers of particles at the top channel inlet (red bar), center channel outlet (blue bar), and top channel outlet (pink bar) are calculated from the NTA profiles. (c) SEM image of the isolated EVs loaded on a cellulose filter membrane.

EV isolation was conducted using ExoSMP with 30 and 100 nm membrane filters. Isolation efficacy was studied and compared under sample flow rates of 5, 10, and 20 *μ*l/min. The size distribution and total number of EVs were measured by NTA and the averaged results of more than three times the measurements were plotted. As shown in Fig. 3(a), the original sample solution (red line) exhibited a broad size distribution, with two specific peaks at approximately 108 and 154 nm representing two major EV subgroups: sEVs and mEVs. At the sample flow rate of 10 *μ*l/min, the particle size distribution acquired from the center outlet (blue line) displayed a narrow range, between 50 and 150 nm, with a single peak at approximately 99 nm; this is consistent with the NTA results from the sample solution and predicted size of EVs based on the pore sizes of the nanomembrane filters. The peak concentration of sEVs in the center channel outlet solution was slightly higher than that of the sample solution. One possible reason could be that the total volume of the collected center outlet solution was slightly less than that of the sample solution (0.5 ml). Moreover, a slight shift in the sEV distribution peaks acquired from the center channel outlet and original sample solutions was observed, which can be attributed to the detection limits of the NTA resolution due to the heterogeneity of the samples. The top channel outlet solution (pink line) exhibited a size distribution between approximately 100 and 280 nm with a single peak at about 156 nm, which corresponded to the other major subgroup of microvesicles. This result is also coincident with the particle distribution from the original sample solution. The total number of the particles in the top channel inlet (red bar), center channel outlet (blue bar), and top channel outlet (pink bar) solutions were 1.17 × 108 per ml, 2.26 × 108 per ml, and 7.95 × 108 per ml, respectively, as shown in Fig. 3(b). The total number of particles collected from both the center and top channel outlets as a fraction of those in the sample solution was 87.2%, suggesting that ExoSMP achieved a high sample yield with minor loss during the isolation process. The sample loss could be due to particles remaining in the microchannels or tubing that were not transported to the outlets. The morphology and size of the isolated EVs were confirmed and examined by SEM, as shown in Figs. 3(c) and Fig. S2 in the supplementary material.

Significant variations in the size distribution profiles were observed under different sample flow rates, given the 0.5 ml volume of sample solution. At the sample flow rate of 20 *μ*l/min, the total number of EVs collected in the center outlet solution was too low to be accurately measured by the NTA. The size distribution of the particles collected from the top outlet (see Fig. S3 in the supplementary material) was analyzed. It showed a similar broad size distribution with two peaks at 120 and 155 nm, which represented the sEV and mEV subgroups. The size distribution of the mEVs exhibited a profile close to that of the original sample solution, while the size distribution of the sEVs showed a low level of EV recovery, indicating that most of the particles were guided to the top outlet channel due to the fast sample flow rate.

**FIG. 4.**
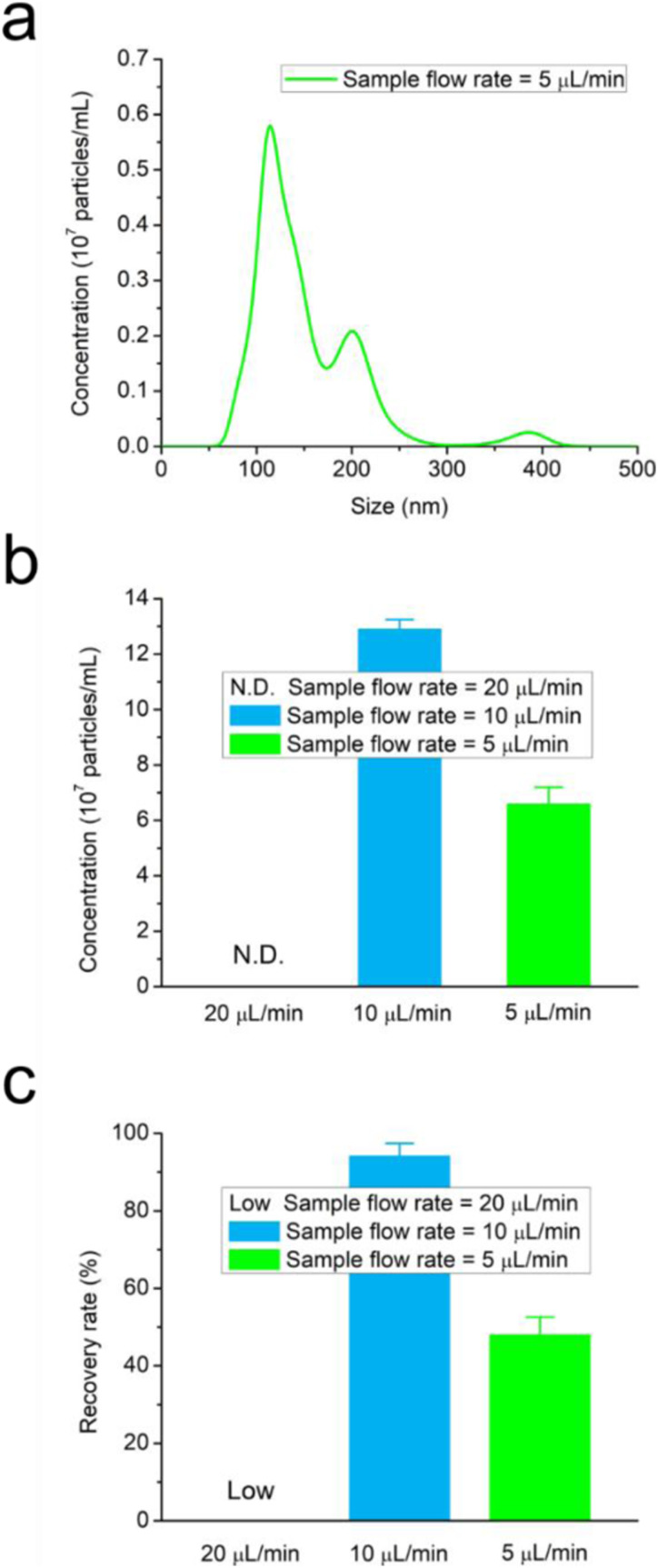
Isolation of EVs from cancer cell derived EVs at different sample flow rates. (a) The size distribution of the isolated EVs collected in the center channel outlet (green line) at the sample flow rates of 5 *μ*l/min. (b) The numbers of particles with dimensions from 30 to 100 nm collected from the center channel outlet solution at different flow rates. ND refers to not detectable. (c) Recovery rates of the EVs with dimensions from 30 to 100 nm determined based on the NTA data.

The size distribution of the particles collected from the center channel outlet (green line) at a sample flow rate of 5 *μ*l/min is shown in Fig. 4(a). A broad size distribution was observed between 50 and 400 nm with two peaks at 113 and 202 nm representing the sEVs and mEVs. The peak at 202 nm indicates that EVs larger than the pore size (100 nm) of the first membrane filter were collected in the center channel outlet. Particles took more residual time to pass through the nanomembrane filter area at this sample flow rate. This phenomenon can be explained by the deformability of EVs through the nanomembranes. EVs consist of soft and flexible membranous lipid structures, which enables them to pass through the membrane filter even if their diameter exceeds the physical size limitation of the pores.^41,42^ With a long residual time, some of the large vesicles (>100 nm) may have had enough time to deform and penetrate through the nanomembrane filter with a pore size of 100 nm, resulting in the peak shift corresponding to sEVs and a broad distribution of mEVs. Coincidently, some small particles with diameters between 30 and 100 nm might also have had sufficient time to pass through the 30 nm membrane filter, thus reducing the number of sEVs recovered from the sample solution. As shown in Fig. 4(b), the total number of particles with dimensions between 30 and 100 nm isolated at the sample flow rate of 10 *μ*l/min was calculated to be 1.29 × 10^8 ^per ml. This was much higher than that of the sample flow rate of 5 *μ*l/min, which was calculated to be 6.58 × 10^7 ^per ml. The recovery rate was defined as a fraction of the total number of particles with dimensions between 30 and 100 nm that were recovered from the center channel solution and total number of particles in the original sample solution. The purity is defined as the fraction of the isolated particles among the collected particles of all sizes in the center solution. As shown in Fig. 4(c), the recovery rate at a sample flow rate of 20 *μ*l/min was low. From our calculations, at a sample flow rate of 10 *μ*l/min, the total number of particles ranging from 30 to 100 nm collected in the center channel outlet solution and the number of particles with the same dimension range collected in the sample solution were 1.29 × 10^8 ^per ml and 1.37 × 10^8 ^per ml, respectively. The recovery rate in this situation was calculated as 94.2%, with a very small standard deviation of 3.2%. The results show that the EVs were isolated from the cancer cell culture media with high rates of recovery and reproducibility at the optimal sample flow rate. We also calculated that the purity of the isolated EVs ranging from 30 to 100 nm was estimated to be 56.3%, In the meanwhile, we calculated the recovery rate and the purity of the isolated EVs ranging from 30 to 110 nm to be 86.9% and 90.7%, respectively. The results implied that of a large proportion of the isolated EVs have size ranging from 100 to 110 nm. Similarly, at a sample flow rate of 5 *μ*l/min, the total number of particles ranging from 30 to 100 nm collected in the center channel outlet solution was 6.58 × 10^7 ^per ml, and the recovery rate was calculated as 48.0%. Overall, the proposed microfluidic platform showed a high rate of recovery of 94.2%, at the optimum sample flow rate for particles ranging from 30 to 100 nm, while also offering the advantage of a short processing time (less than 1 h).

**FIG. 5.**
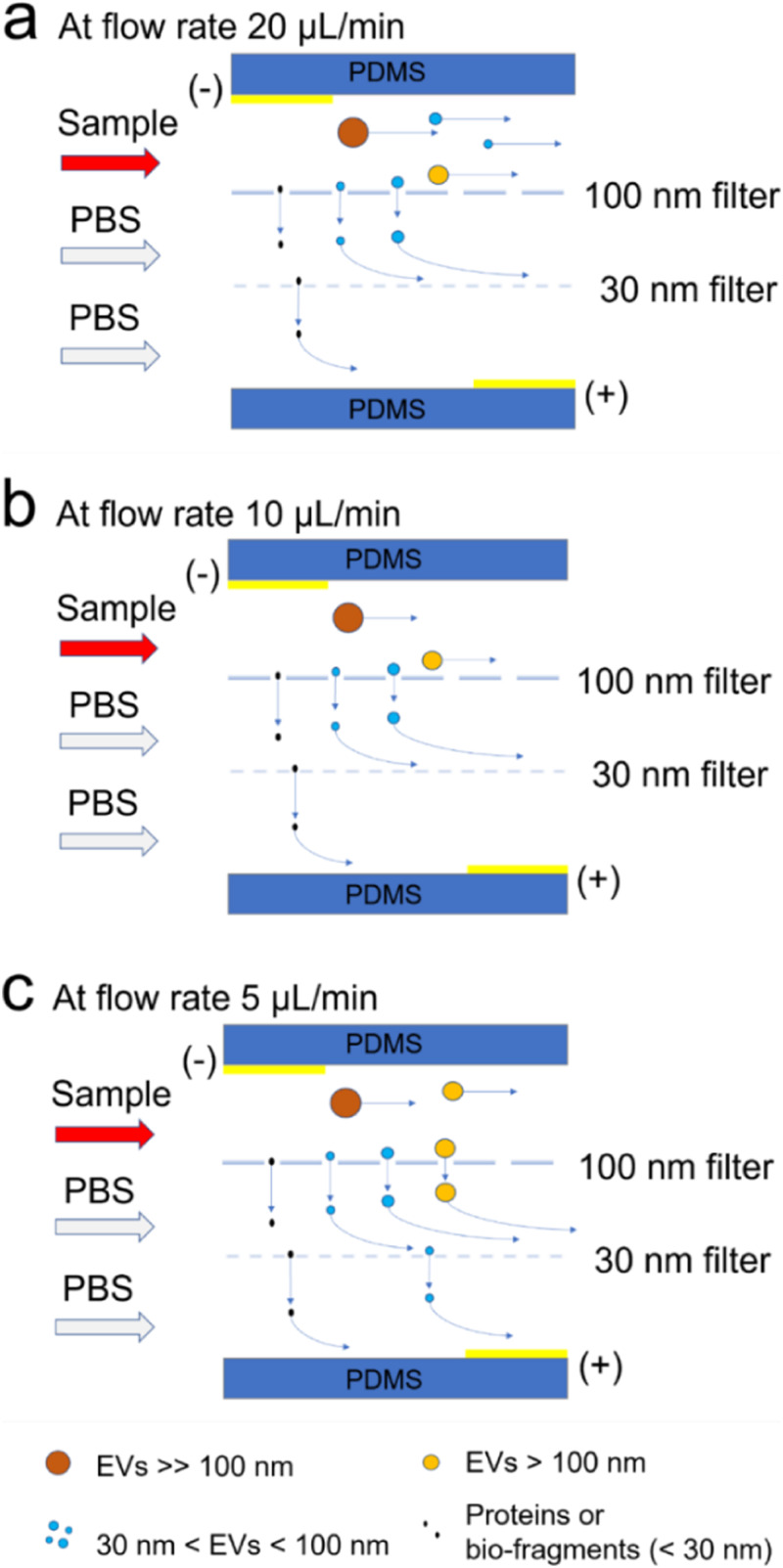
Schematic side view diagrams of the EV isolation processes at different sample flow rates. (a) 20 *μ*l/min (b) 10 *μ*l/min and (c) 5 *μ*l/min sample flow rates. Schematic particle trajectories are labeled in blue arrows.

The schematic diagram of the isolation process at a sample flow rate of 20 *μ*l/min is shown in Fig. 5(a). In this case, the EVs had less residual time to pass through the nanomembrane filters and most of the particles were quickly transported to the top channel outlet, which led to a low EV recovery. In contrast, the isolation mechanism at a sample flow rate of 10 *μ*l/min is shown in Fig. 5(b). ExoSMP achieved a high particle recovery rate at the optimized sample flow rate. The schematic diagram of the isolation process is shown in Fig. 5(c) at a sample flow rate of 5 *μ*l/min. In this situation, the EVs took more residual time to pass through the nanomembranes due to their soft and flexible membranous structures, which led to the peak corresponding to microvesicles, and thus reduced the EV recovery compared to that at a sample flow rate of 10 *μ*l/min.

### EV subpopulation isolation with different nanomembrane combinations

**FIG. 6.**
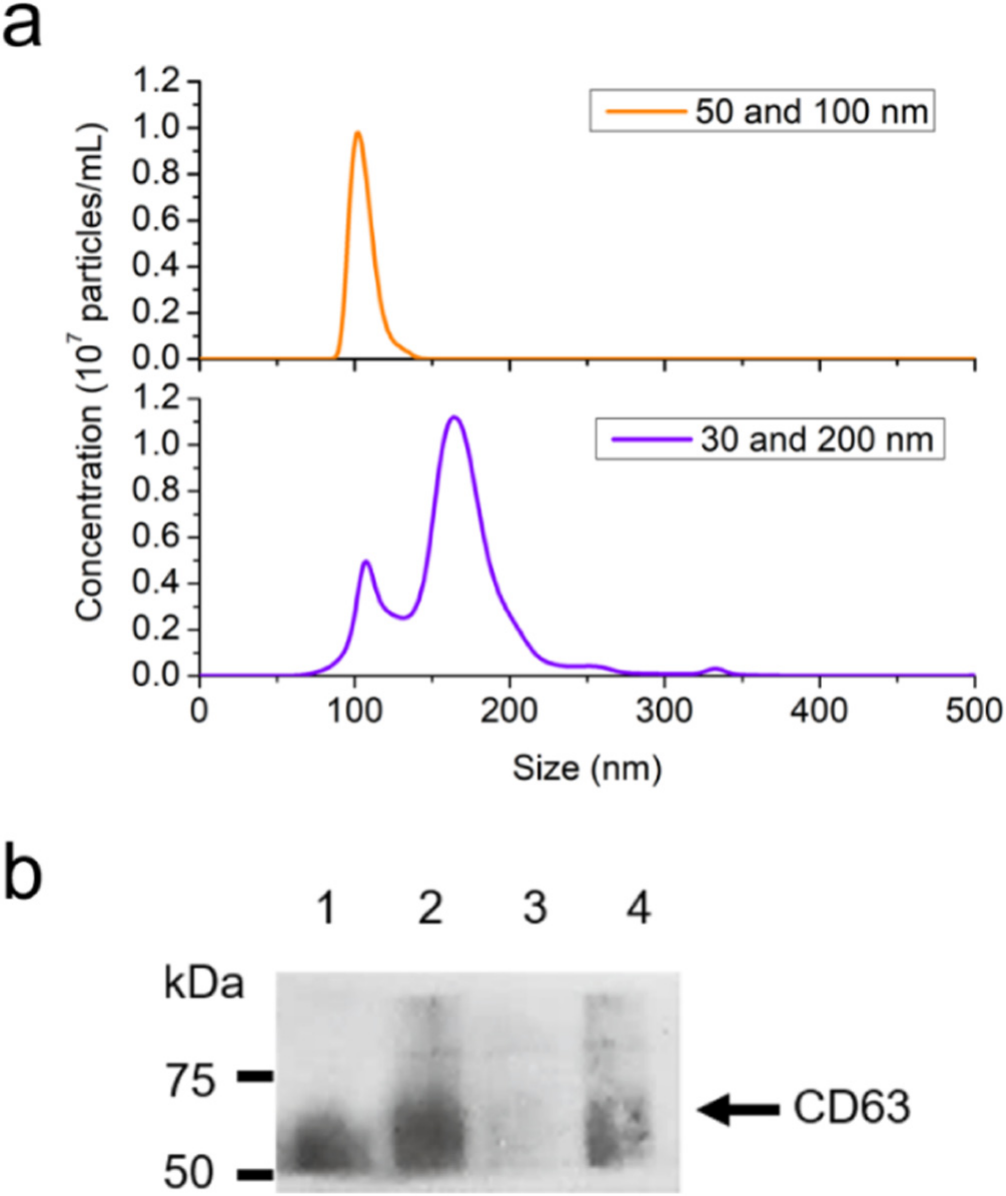
Isolation of sEV subpopulations and EV subgroups with ExoSMP and different pore sizes. (a) The size distributions of the isolated EVs with different nanoporous membrane combinations by NTA. (b) Western blot analysis of EV biomarker CD63 in the isolated EVs with different nanomembrane combinations: (1) 50 and 100 nm, (2) 30 and 100 nm, (3) 30 to 200 nm, and (4) 30 and 50 nm.

We further demonstrated the microfluidic platform as a modular unit for sEV subpopulation and EV subgroup isolation with tunable size groups. The ExoSMP technique was used to separate subpopulations of sEVs and subgroups of EVs simply by altering the pore sizes of the nanomembrane filters. Size-selective isolation was investigated with different nanomembrane combinations: 50 and 100 nm, 30 and 50 nm, and 30 and 200 nm. The isolation process and parameters were same as those of the sEV isolation with 30 and 100 nm membrane filters. The size distributions of the isolated EVs from the nanomembrane combinations of 50 and 100 nm and 30 and 200 nm were acquired by NTA, as shown in Fig. 6(a). The sEV subpopulation isolated by 50 and 100 nm nanomembrane filters displayed a narrow size distribution with a single peak at 100 nm, which corresponded to the major EV subgroup of sEVs. This size distribution profile was similar to that of sEVs isolated by nanomembranes with 30 and 100 nm pore sizes. The EV subgroup isolated by 30 and 200 nm nanomembrane filters exhibited a broad size distribution, between 50 and 400 nm. Single peaks at about 107 nm and 163 nm corresponded to the two major EV subgroups of sEVs and mEVs. The size distribution displayed a profile similar to that of the original sample solution. A valley between two peaks at 132 nm was consistent with that of the original sample solution at 130 nm. A small peak at about 330 nm indicated that there might be a small amount of microvesicles penetrating through the 200 nm membrane filter, or some EV aggregation. The size distribution of the isolated EVs collected from the device with 30 and 50 nm membrane filters was not applicable, which was mainly attributable to the detection limit of NTA for heterogeneous samples of small dimensions. For each size group, the EV transmembrane protein CD63 was studied by western blot analysis, as shown in Fig. 6(b). The isolated exosomal biomarker was confirmed by smear patterns, which were observed for α-CD63, as expected.^22^ The EVs were isolated with the following nanomembrane combinations: 30 and 100 nm, 30 and 200 nm, and 30 and 50 nm; they showed broad smear patterns ranging from 50 to above 75 kDa, while only the isolated EVs with the 50 and 100 nm nanomembrane combination showed narrow smear patterns in the range of 50–75 kDa. It is of note that the light smear patterns for the isolated EVs with the 30 and 200 nanomembrane combination were due to a low EV recovery, as shown by the size distribution measured by the NTA result [see Fig. 6(a)].

## CONCLUSION

In this work, we developed and demonstrated a size-selective microfluidic platform (ExoSMP) for automated, consistent, and reliable EV isolation. This unique platform offers an enhanced approach to the isolation of EV subgroups and sEV subpopulations, along with the additional advantage of being label-free, low-cost, and featuring a short processing time (<1 h), and convenient integration with downstream analysis. This platform demonstrated a high recovery rate of 94.2% and reproducibility (a low standard deviation) from cancer cell culture media samples with an optimal sample flow rate. The size-selective isolation of EVs can easily be controlled by altering the pore sizes of the nanomembrane combinations. We further utilized ExoSMP with various combinations of nanomembrane pore sizes to demonstrate the isolation of EV subgroups and investigate sEV subpopulations of various size groups. The western blot analysis suggested the evidence of CD63 biomarker in the subgroups of the EVs. This improved technique will serve as a precise clinical tool for isolating EVs and addressing the heterogenicity of EV subgroups. Additionally, with its efficient size-based isolation of EV subpopulations, ExoSMP shows broad promise for investigating the role of EVs in various point-of-care applications in disease monitoring, medical diagnosis, and drug delivery.

## EXPERIMENTAL

### Device fabrication and assembly

The microfluidic device consists of three polydimethylsiloxane (PDMS) microchannels and two track-etched nanoporous polycarbonate (PC) membranes (Whatman, GE Healthcare Life Sciences). Each microfluidics layer was designed by computer-aided design (CAD) software. The master mold for each layer was printed by a 3D printer (Envision). The printed molds were exposed to ultraviolet (UV) light for 5 min and cured at 65 °C for at least 24 h.

The microchannel layers were fabricated by soft lithography (as described previously) with some modifications.^43,44^ A Sylgard 184 Silicone Elastomer (Dow Corning) curing agent and base liquid were thoroughly mixed at a weight ratio of 1:10, followed by a degassing process in a desiccator with a mechanical vacuum pump to remove any air bubbles and ensure a thorough mix of the two liquids. Then, a prepolymer mixture was cast on top of the master molds and cured at 65 °C for 4 h. After curing, the PDMS replicates were peeled off from the master molds. The top layer was punched with three inlets and three outlets and the center layer was punched with two holes to connect the bottom microchannel inlet and outlet. A large through-hole was also punched in the center layer.

In the bottom/center layer bonding process, the channel side of the bottom layer and flat side of the center layer were treated with oxygen plasma for 2 min. A piece of PC membrane filter was sandwiched between these two layers. A uniform pressure was applied to the top of the device for 60 s. Then, the device was baked in the oven at 65 °C for 2 h. After baking, the device was taken out for a second bonding process with the top layer. Another piece of PC nanomembrane was sandwiched between the bonded bottom/center layers and the top layer. After applying the appropriate pressure on top of the device to form pre-bonding, the three-layer device was baked at 65 °C for 2 h to improve bonding quality. Microfluidic devices with four groups of nanomembrane combinations were fabricated. The pore-size combinations were (1) 50 and 100 nm, (2) 30 and 100 nm, (3) 30 to 200 nm, and (4) 30 and 50 nm.

After removing the device from the oven, six pieces of tubing (Tygon 3350) were cut at the same length and connected to the inlets and outlets of the device. The free ends of the inlet tubing were connected to syringes, while the free ends of the outlet tubing were connected to containers that collected the separated solutions. Gold wires were employed as electrodes and pinned at the same position of the top inlet and bottom outlet tubing. A well-mixed epoxy was applied around each tube to prevent any leakage from the electrode points. Then, the device was baked at 65 °C for 30 min to completely cure the epoxy glue.

### Cell culture

The MDA-MB231 cell line was obtained from the American Type Culture Collection (Manassas, VA). It was independently validated using STR DNA fingerprinting at MD Anderson Cancer Center. Tests for mycoplasma contamination were negative. Cells were grown in a Dulbecco's Modified Eagle Medium, or DMEM, supplemented with 10% fetal bovine serum at 37 °C in 5% CO_2_.

### EV sample preparation from the cell culture media

Cells were cultured for 72 h. EVs were collected from their culture media after sequential ultracentrifugation as described previously.^45^ In brief, cells supernatants were collected and centrifuged at 300 g for 10 min. The supernatants were collected for centrifugation at 2000 g for 10 min, then collected again for another centrifugation at 10 000 g for 30 min. Finally, the supernatants were ultra-centrifuged at 100 000 g to pellet the EVs. The pellets were washed in a large volume of PBS to eliminate contaminating proteins and centrifuged one last time at the same high speed. The final pellets containing EVs were re-suspended in PBS and stored at −80 °C. The total EV protein concentrations were determined using a bicinchoninic acid (BCA) protein assay.

### Experiment procedure

The initial sample cell culture media solution was prepared by diluting 1 μ stock EV solution with a PBS solution, for a total volume of 1 ml. To isolate sEVs with 30 and 100 nm membrane filters at different sample flow rates, 0.5 ml of the initial solution was pumped into the top microchannel at flow rates of 20, 10, and 5 *μ*l/min, and the PBS buffer solution was pumped into the center and bottom microchannels at flow rates of 40 and 5 *μ*l/min. Gold wires were connected to the positive and negative ends of a power supply and the applied voltage was set at 500 V (the electric field was calculated to be approximately 20 V/cm). The outlets of the tubing were connected to containers to collect the solutions containing EVs. The total processing time was 50 min (less than 1 h). The rest of the initial and isolated solutions were kept in a refrigerator at 4 °C for a subsequent quantification analysis. The experiment setups were the same for the EV subgroup isolations using nanomembrane combinations of (1) 50 and 100 nm, (2) 30 and 100 nm, (3) 30 to 200 nm, and (4) 30 and 50 nm.

### Nanoparticle tracking analysis

The size distribution and total number of the particles in the original sample and isolated solutions were measured by NTA. Before measurement, the sample solution was diluted 10 times to fit the suggested particle concentration range for the instrument. The center channel solution was also diluted 2.5 times. The top outlet channel solution was also diluted 10 times before characterization. The measurement was conducted at room temperature using a NanoSight LM10 system (Malvern, Worcestershire, UK) with an emitting laser λ = 405 nm. Samples were manually introduced from a syringe and video images were recorded and analyzed by NTA software version 3.2. Particle distributions were measured at least three times. The average results of the particle size distributions were then plotted; the dilution factors were considered when plotting the figures and calculating the total number of particles.

### Scanning electron microscopy

Scanning electron microscopy (SEM) image of the EVs was acquired by a Tescan scanning electron microscope. The isolated EVs were first filtered through a cellulose membrane (Whatman) to remove the PBS buffer, followed by washing with serial concentrations of ethanol to fully dehydrate the isolated EVs. The concentrations of ethanol were set at 50%, 60%, 70%, 80%, 90%, 95%, and 100%. Each washing was conducted three times. A 5-nm-thick Pt/Pd thin film was deposited on the surface of the isolated EVs on the filter membrane to increase the sample's conductivity for SEM imaging. The pore size distributions of the PC nanoporous membranes were also studied by SEM. The nanoporous membranes with different pore sizes (30, 50, 100, and 200 nm) were cut into small pieces and sputtered with a 5-nm-thick Pt/Pd film for SEM imaging.

### Western blot analysis

EV samples were re-suspended in a 5× sodium dodecyl sulfate (SDS) loading buffer (0.25M Tris-HCl pH 6.8, 10% SDS, 50% glycerol, 0.5M dithiothreitol, 0.25% bromophenol blue) and boiled at 95 °C for 10 min. Then, the samples were separated by 10% SDS-polyacrylamide gel electrophoresis (100 V, 2 h) and transferred to polyvinylidene difluoride (PVDF) membranes (90 V, 2 h). The membranes were blocked in a 5% milk solution at room temperature (RT) for 1 h and incubated with the CD63 primary antibody (BioLegend, mouse anti-human) diluted with the blocking solution overnight at 4 °C. After washing three times with TBST, the membranes were incubated with a secondary antibody (anti-mouse IgG-HRP) at RT for 1 h. After washing the membrane three times with tris-buffered saline with Tween-20 (TBST), signals were detected using a chemiluminescent substrate (Thermo Fisher).

## SUPPLEMENTARY MATERIAL

See the supplementary material for the detailed design of the microchannels, SEM images of the isolated EVs, and the size distribution of particles collected from the top channel outlet at a sample flow rate of 20 *μ*l/min.

## DATA AVAILABILITY

The data that support the findings of this study are available within the article and its supplementary material.
